# Effect of dexamethasone on carrageenin-induced inflammation in the lung

**DOI:** 10.1155/S0962935195000445

**Published:** 1995-07

**Authors:** S. F. Smith, A. Benjamin, A. Dewar, M. Sheppard, B. Fox, T. Smith, A. Guz, T. D. Tetley

**Affiliations:** 1 Department of Medicine Charing Cross and Westminster Medical School Fulham Palace Road London W6 8RF UK; 2 Department of Histopathology Charing Cross and Westminster Medical School Fulham Palace Road London W6 8RF UK; 3 Electron Microscopy Unit Royal Brompton Hospital Sydney Street London SW3 6NP UK; 4 Department of Histopathology Royal Brompton Hospital Sydney Street London SW3 6NP UK

## Abstract

To study the anti-inflammatory mechanisms of glucocorticoids, we have compared the effects of intratracheal carrageenin (2.5 mg) on control rats and those in which inflammation was subdued by prior dexamethasone treatment (10 mg/l in drinking water). Inflammation was maximal 48 h post-carrageenin. After dexamethasone, carrageenin caused tittle inflammation or oedema (wet lung (mg), n = 6, mean ± S.E.M.; control, 995 ± 51; carrageenin + dexamethasone, 1144 ± 83; compared with carrageenin alone, 1881 ± 198), but rats had more lung lavage neutrophils than those given carrageenin alone (PMN × 10^6^ /lung, mean ± S.E.M.; control, 0.055 ± 0.003; carrageenin + dexamethasone, 8.54 ± 1.52; compared with carrageenin alone, 6.30 ± 1.71). Proteolysis and partial inactivation of the anti-inflammatory mediator, lipocortin 1 (Lcl), in carrageenin-instilled rats was offset in those also given dexamethasone, by increased Lc1 levels (intact Lc1 ng/ml lavage fluid, n = 4, mean ± S.E.M.; control 24 ± 6; carrageenin 15 ± 4; carrageenin + dexamethasone, 40 ± 15). Maintenance of sufficient intact (fully active) extracellular Lc1 may contribute to the actions of glucocorticoids.


Mediators of Inflammation 4, 273–281 (1995)

### Effect of dexamethasone on carrageenin-induced inflammation in the lung

S. F. Smith,[1,CA] A. Benjamin,[1] A. Dewar,[3] M. Sheppard,[4] B. Fox,[2] T. Smith,[1] A. Guz[1] and T. D. Tetley[1]

Departments of [1]Medicine and [2]Histopathology, Charing Cross and Westminster Medical School, Fulham Palace Road, London, W6 8RF; [3]Electron Microscopy Unit and [4]Department of Histopathology, Royal Brompton Hospital, Sydney Street, London, SW3 6NP, UK

[CA]Corresponding Author

**To study the anti-inflammatory mechanisms of glucocorticoids, we have compared the effects of intratracheal carrageenin (2.5 mg) on control rats and those in which inflammation was subdued by prior dexamethasone treatment (10 mg/l in drinking water). Inflammation was maximal 48 h post-carrageenin. After dexamethasone, carrageenin caused little inflammation or oedema (wet lung (mg), *n* = 6, mean ± S.E.M.; control, 995 ± 51; carrageenin + dexamethasone, 1144 ± 83; compared with carrageenin alone, 1881 ± 198), but rats had more lung lavage neutrophils than those given carrageenin alone (PMN × $10^6$/lung, mean ± S.E.M.; control, 0.055 ± 0.003; carrageenin + dexamethasone, 8.54 ± 1.52; compared with carrageenin alone, 6.30 ± 1.71). Proteolysis and partial inactivation of the anti-inflammatory mediator, lipocortin 1 (Lc1), in carrageenin-instilled rats was offset in those also given dexamethasone, by increased Lc1 levels (intact Lc1 ng/ml lavage fluid, *n* = 4, mean ± S.E.M.; control 24 ± 6; carrageenin 15 ± 4; carrageenin + dexamethasone, 40 ± 15). Maintenance of sufficient intact (fully active) extracellular Lc1 may contribute to the actions of glucocorticoids.**

**Key words:** Annexin 1, Bronchoalveolar lavage fluid, Carrageenin, Glucocorticoid, Lipocortin 1, Polymorphonuclear neutrophil, Pneumonitis

### Introduction

Inflammation is a complex response to tissue damage, often due to microbial invasion, involving the release and interactions of many mediators including cytokines, proteinases, eicosanoids and oxidants[1] from resident and newly infiltrating cells. It is a particularly important defensive mechanism in the airways and lungs, which are constantly exposed to inhaled biological, chemical and physical insults.

The endogenous mechanisms regulating inflammation in the lung and in other tissues are incompletely understood. However, studies on rodents have shown that prior surgical or chemical adrenalectomy exacerbates the inflammatory response to any given stimulus,[2,3] an effect which can be ameliorated by glucocorticoid replacement therapy, indicating that physiological levels of endogenous circulating glucocorticoid normally regulate inflammatory processes. Glucocorticoids operate via specific receptors at the transcriptional level to alter the expression and activities of specific gene products (reviewed in Reference 4). They may also exert indirect effects on inflammatory mediators, for example by altering the expression of their receptors.[5]

Lipocortin 1 (Lc1), a member of the annexin family,[6] is glucorticoid-inducible *in vivo*[7–9] and has anti-inflammatory activity,[10] expression of which is thought to require binding of Lc1 to specific sites on cell surfaces.[11] It is normally present in the lung, both intracellularly, in a variety of different cell types[12] and extracellularly, in the epithelial lining fluid.[13,14] Glucocorticoids increase the concentrations of Lc1 in a variety of lung cells, including alveolar epithelial cells[15] and alveolar macrophages,[16] and may promote its release onto the cell surface and/or into epithelial lining fluid[13,17,18] where it would be available for receptor binding. Thus, Lc1 could play a pivotal role in the anti-inflammatory actions of glucocorticoids in the lung, but currently its contribution is unclear.

In this paper, we describe a model of carrageenin-induced pneumonitis which we have characterized in control animals and in those in which the inflammatory response was subdued by prior and concurrent treatment with exogenous glucocorticoid. We have also quantified extracellular Lc1 to assess its putative contribution to the actions of dexamethasone.

© 1995 Rapid Science Publishers

### Materials and Methods

*Experimental model of inflammation:*

*Animals.* Groups of mature CFY rats, bred in-house and weighing between 180 and 220 g at the beginning of the experiment, were housed in pairs and given unlimited access to food and water or water containing dexamethasone (see below). At the end of the appropriate experimental period, all animals were anaesthetized with halothane then sacrificed by sodium thiopentone overdose.

*Carrageenin treatment.* Animals undergoing carrageenin instillation were lightly anaesthetized with halothane and 2.5 mg carrageenin/0.5 ml sterile 0.15 M NaCl (lambda-carrageenin (Type IV), Sigma Chemical Co., Poole, UK) was instilled into the lung via the trachea using a previously described method,[19] except that the suspension was instilled through a flexible 3FG catheter (Portex Ltd.) which was introduced through the mouth and between the vocal cords.

*Dexamethasone treatment.* Dexamethasone (dexamethasone sodium phosphate, David Bull Laboratories, Warwick, UK) was administered in drinking water at a dose of 10 mg/l. Average consumption of fluid was approximately 10% of body weight per day. This represented a dose of approximately 1 mg dexamethasone/kg body weight/day.

*Experimental design.* The various experimental groups are described in more detail below.
*(a) Controls (CON).* Control animals were required to illustrate and define the physiological norm and were, therefore, entirely untreated. They were not sham-instilled because the main purpose of the experiment was to establish the effect of dexamethasone on the inflamed lung, not to establish the contribution of saline to the inflammatory response provoked by carrageenin instillation.
*(b) Carrageenin (CARR).* These animals were used to determine the effect of carrageenin on the lung. Animals were instilled with carrageenin as described above and sacrificed 48 h later.
*(c) Carrageenin and dexamethasone (CARR+ DEX).* This group of rats was used to examine the effect of dexamethasone treatment on the carrageenin-inflamed lung. The animals received dexamethasone in drinking water for 48 h prior to carrageenin exposure. They were then exposed to intratracheal carrageenin and continued to receive dexamethasone for a further 48 h. At this time, they were sacrificed as described above for comparison with rats exposed to carrageenin alone.
*(d) Dexamethasone (DEX).* These animals were used to determine the effect of dexamethasone on the normal lung over the entire time course of the experiment. They received the same dose of oral dexamethasone for the same length of time (96 h) as the CARR + DEX treated rats, but were not exposed to carrageenin.
*(e) Baseline dexamethasone (BASE DEX).* These animals were needed to establish the effect of dexamethasone pretreatment on the lungs of rats in the CARR + DEX experimental group prior to carrageenin administration. Thus, they were dosed with dexamethasone for 48 h then sacrificed as described above.

*Pilot studies.* As well as the carrageenin dosing regimen and the experimental time course described above, we performed pilot studies to assess the degree of pneumonitis in animals killed (a) 48 h after a high intratracheal (i.t.) dose of carrageenin (5 mg/0.5 ml NaCl i.t.); (b) 4 h after the standard dose of carrageenin (2.5 mg/0.5 ml NaCl i.t.); and (c) 96 h after the standard dose of carrageenin. Inflammation was assessed by histopathological examination.

*Assessment of the inflammatory response to carrageenin with and without dexamethasone:*

*Light microscopy.* At least two animals were assigned to each of the experimental groups described above. Following treatment and sacrifice according to the protocols described above, the lungs were excised and fully inflated under constant pressure (25 cm) with formaldehyde (4% v/v in phosphate buffered saline) which was instilled via the trachea. Following fixation, the lungs were prepared using standard histological procedures and stained with haematoxylin and eosin. They were examined blind.

*Bronchoalveolar lavage and venous blood.* Four rats were assigned to each group. Following death, the pulmonary circulation was perfused *in situ* with 0.15 M NaCl via the right ventricle. When the lungs were free of blood, they were removed from the thoracic cavity and lavaged via the trachea with 6 × 8 ml portions of 0.15 M NaCl.[19] The recovered lavage fluid was pooled and the volume recorded, a sample removed for total cell count and differential cell profile by Wright–Giemsa staining, then the remaining fluid was processed as described previously.[20]

Venous blood samples (0.2 ml) were taken from each animal for differential white blood cell analysis before the beginning of the experiment, at the time of carrageenin instillation and at the time of sacrifice. Smears were made of each blood sample, allowed to air dry and stained with

Wright–Geimsa stain. The proportion of polymorphonuclear neutrophils (PMN) and mononuclear cells was determined by light microscopy. Approximately 150 cells were counted per smear except in dexamethasone-treated animals in which circulating leukocytes were sparse.

*Electron microscopy.* The experiment was performed on two rats per treatment group as described for the light microscopy study, except that the lungs were inflated with 2% paraformaldehyde and 2.5% glutaraldehyde in 0.1 M sodium cacodylate buffer, pH 7.4. After 24–72 h, this fixative was replaced with 50 mM sodium cacodylate buffer, pH 7.4, containing 7.5% sucrose. The lungs were encoded and stored at 4°C until prepared for electron microscopy using standard techniques. They were examined blind.

*Assessment of oedema by wet and dry lung weight measurements.* Animals (six or seven per group) were treated as described above. Following sacrifice, the lungs were excised and lavaged as described above.[19] The major airways were removed, then the parenchymal tissue was blotted to remove excess moisture and weighed (wet weight). The weighed tissue was then minced and homogenized in a Waring blender with an equal mass of distilled water to give a 50% (w/w) homogenate which was divided into two equal portions. One half was placed in a pre-weighed tube and the dry weight was determined by heating to 100°C and re-weighing daily until no further weight loss occurred. The wet weight of 1 ml of autologous blood was measured, then its dry weight was determined by the same method.

The remaining half of each lung homogenate was centrifuged at 30 000 × $g$ at 4°C for 1 h to remove debris. The resulting supernatant was assayed in duplicate for haemoglobin by the cyanmethaemoglobin method.[21] The haemoglobin content of a sample of autologous whole blood from each rat was also quantified by the cyanmethaemoglobin method. The haematocrit was measured by centrifuging duplicate capillary tubes of venous blood in a Hawksley microcentrifuge for 2 min and reading the percentage of cells on a calibrated scale.

The contribution of blood to the wet and dry weights of each lung could then be calculated as described previously,[22] except that haemoglobin was used as a marker of blood. It was then possible to determine the blood-free lung water and wet and dry lung weights. The haemoglobin content of bronchoalveolar lavage, both fluid and cells, was also assessed but was below the limits of detection in all samples.

*Analysis of extracellular lipocortin 1 in bronchoalveolar lavage fluid.* The acellular bronchoalveolar lavage fluid (BALF) samples analysed in this part of the experiment were those collected as described in a previous section. The cellular Lc1 content was not determined in this study. Extracellular Lc1 levels were measured using a previously described[23] competitive enzyme-linked immunosorbent assay (ELISA) modified as follows. Gelatin (2% w/v) was used as a blocking agent, wells were coated with 100 µl Lc1 (1 µg/ml) and the polyclonal anti-Lc1 antibody was used at a dilution of 1:30 000 in phosphate buffered saline containing 0.5% Tween.

Samples of BALF were concentrated 7.5-fold by centrifugation in Centricon units with a nominal molecular weight cut-off of 2 000 kDa. Concentrates were separated by SDS–polyacrylamide gel electrophoresis, followed by Western blotting and staining for Lc1 as described previously.[14] The relative proportions of intact and proteolysed Lc1 were determined by scanning densitometry as described previously.[14]

*Statistics:* Where appropriate, statistical analysis was performed using a Wilcoxon rank sum test for unpaired data. The level of significance was $p < 0.05$.

### Results

*Macroscopic appearance:* Lungs from rats given carrageenin alone (CARR) appeared mottled with hard, lumpy areas. The lungs of all other animals were macroscopically normal. The pleura appeared normal in all animals.

*Histopathology:* In pilot studies, no changes were seen at the light microscopic level 4 h after carrageenin instillation (Fig. 1b). The peak effect of carrageenin (CARR) was observed 48 h after instillation and consisted of large areas of interstitial and intra-alveolar pneumonitis, with alveolar wall thickening and a prominent cellular infiltration (Fig. 1c). By 96 h after instillation, there were fewer cells in airspaces and interstitium, and the alveolar walls were less swollen; the inflammation appeared to be resolving (Fig. 1d). In pilot studies (data not shown) it was found that a dose of 5 mg carrageenin provoked the same inflammatory response at 48 h post-instillation as 2.5 mg which we, therefore, believe produces a maximal effect. Dexamethasone alone (DEX: BASE DEX) had no effect on pulmonary histopathology at any of the time points examined (Fig. 1f). In animals given both glucocorticoid and carrageenin (CARR + DEX), there was little alveolar wall thickening; however, the

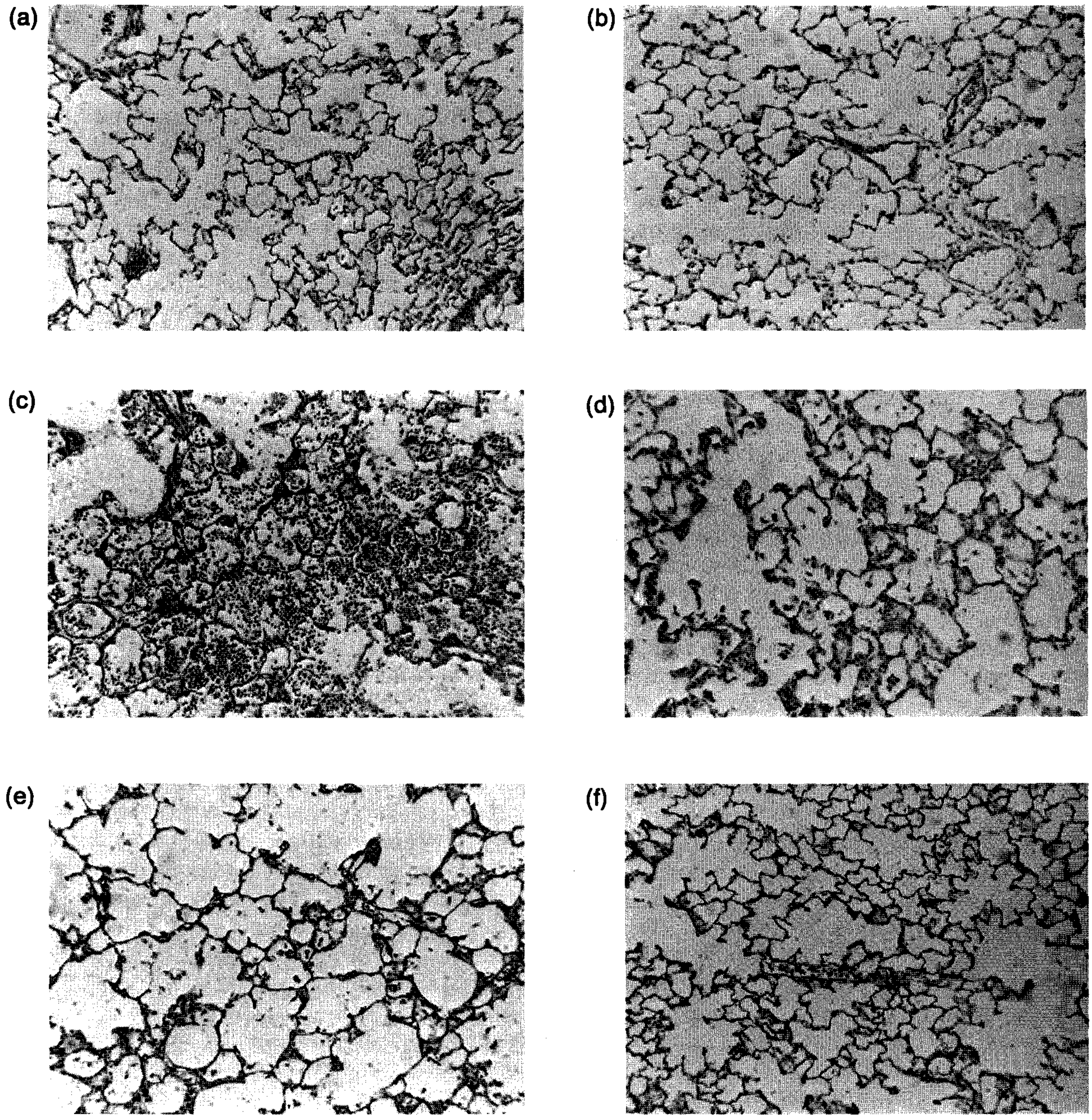

FIG. 1. Light microscopy illustrating the evolution and resolution of the carrageenin-induced pneumonitis and the effect of dexamethasone. All sections are photographed at a magnification of × 38.
(a) Control (CON); (b) 4 h post-instillation of carrageenin; no significant changes seen; (c) 48 h post-instillation of carrageenin (CARR); alveolar wall thickening and marked cellular infiltration; (d) 96 h post-instillation of carrageenin; fewer inflammatory cells in the air- and interstitial spaces and less alveolar wall swelling than at 48 h; (e) dexamethasone-treated at 48 h after carrageenin instillation (CARR+DEX); less alveolar wall swelling and fewer cells in the interstitium and airspaces than in rats given carrageenin alone at the same time after instillation; and (f) dexamethasone alone (DEX); not significantly different to control.

cellular infiltration was not completely suppressed (Fig. 1e).

*Bronchoalveolar lavage cell number and profile and fluid recovery:* The fluid recovery was greater from animals given oral dexamethasone for 48 h prior to sacrifice (BASE DEX, 49 ± 3 ml, mean ± S.E.M.) than from those in any other group (mean ± S.E.M.; CON, 35 ± 3 ml; DEX, 35 ± 4 ml; CARR, 32 ± 5 ml; CARR + DEX, 32 ± 4 ml). The total cell recoveries for each group are shown in Fig. 2. In all but the animals given both carrageenin and dexamethasone (CARR + DEX), the majority of cells in BALF were alveolar macrophages (AM) and the remainder polymorphonuclear neutrophils (PMN). Eosinophils were not detected in any lavage sample and lymphocytes were rare. Instillation of carrageenin

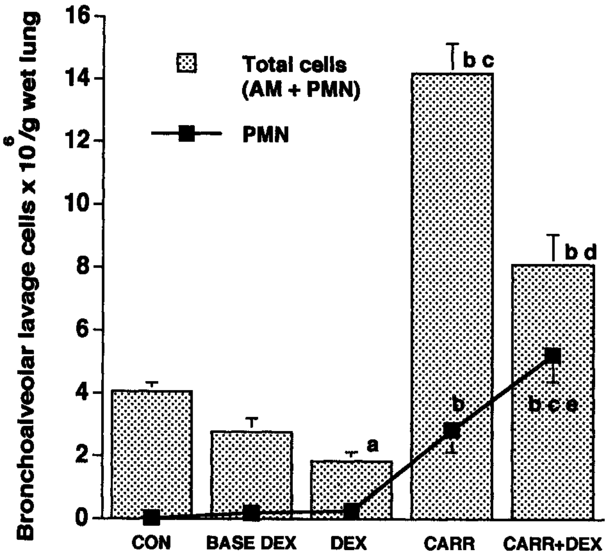

FIG. 2. Effect of treatment on total cell and polymorphonuclear neutrophil numbers in bronchoalveolar lavage fluid. PMN, polymorphonuclear neutrophil; AM, alveolar macrophage; CON, animals receiving no treatment; BASE DEX, animals given dexamethasone (10 mg/l) in drinking water for 48 h prior to sacrifice; DEX, animals given dexamethasone (10 mg/l) in drinking water for 96 h prior to sacrifice; CARR, animals given 2.5 mg carrageenin intratracheally and sacrificed 48 h later; CARR+DEX, animals given dexamethasone for 48 h prior to carrageenin exposure (2.5 mg intratracheally) and for a further 48 h after carrageenin exposure, after which they were sacrificed. a < CON; b > CON; c > DEX; d < CARR; e > CARR; in each case, $p < 0.05$ in a Wilcoxon rank sum test for unpaired data. Data expressed as means ± standard error of mean; $n = 4$. Where no error bar is visible, the standard error is within the diameter of the symbol.

(CARR) was associated with significant increases in the total cell number; the majority of cells in BALF were AM, although there was also a significant increase in the number of PMN (Fig. 2). Dexamethasone treatment for 48 h (BASE DEX) had no effect on cell number or type (Fig. 2). However, after 4 days of dexamethasone (DEX), the total number of cells in BALF was reduced compared with the untreated (CON) animals (Fig. 2). Interestingly, whilst the total number of cells in BALF from animals given carrageenin plus dexamethasone (CARR + DEX) was intermediate between those given either stimulus alone, there were more PMN in their BALF than in that of any other treatment group (Fig. 2), indicating that the dexamethasone had selectively suppressed the AM infiltration. The mean number of AM/g wet tissue following various treatments was as follows: CON, $4.02 \times 10^6$/g; BASE DEX, $2.58 \times 10^6$/g; DEX, $1.58 \times 10^6$/g; CARR, $11.35 \times 10^6$/g; CARR + DEX, $2.87 \times 10^6$/g.

*Circulating white blood cells:* The blood smears indicated that carrageenin had no effect on the differential white blood cell ratios at any time point (CON = 9 ± 2% polymorphs, CARR = 16 ± 3% polymorphs, mean ± S.E.M., $n = 6$ per group, $p > 0.05$). In rats treated with dexamethasone, or carrageenin and dexamethasone, most of the circulating leucocytes were polymorphs (DEX = 74 ± 2%; CARR + DEX = 83 ± 3%). This change in differential count was due to a substantial reduction in the number of circulating mononuclear cells; the number of circulating PMN fell by a lesser amount in response to exogenous glucocorticoid.

*Electron microscopy:* Following carrageenin instillation (CARR), the alveolar spaces contained large numbers of foamy AM, some containing particles (probably carrageenin) in vacuoles (Fig. 3a). PMN were also observed. In addition, the alveolar spaces contained many round bodies, usually devoid of organelles and thus, appearing comparatively electron-lucent (Fig. 3a). Similar structures were associated with some Type II alveolar epithelial cells and high magnification revealed continuity between the organelle-rich cell cytoplasm and the electron-lucent bulge (Fig. 3b), indicating that the bodies probably originated from the Type II cells. Type II cells were present in increased numbers, often several in close proximity (Fig. 3a). Damage to alveolar Type I cells was common (Fig. 3b). Occasionally, lengths of basement membrane denuded of epithelium were seen; these were associated with areas of interstitial oedema (Figs 3c and 3d). There was also evidence of endothelial damage, with shrinking and increased cell density (Fig. 3d). An increase in interstitial collagen was observed.

By comparison, the changes were minor in the lungs of rats given carrageenin plus dexamethasone (CARR + DEX). There was a slight increase in the number of AM compared with the control groups, and occasional swelling of the Type I epithelial cells was seen (Fig. 1; EM not shown). No difference was seen between groups given dexamethasone alone (DEX) and untreated controls (CON), all of which appeared normal (Fig. 1; EM not shown).

*Assessment of oedema by wet and dry lung weights:* In untreated rats, approximately 90% of the total lung weight (excluding blood) was water. Carrageenin treatment alone (CARR) resulted in significant increases in lung water and dry lung weight compared with the control and those given glucocorticoid alone (DEX and BASE DEX; Table 1). The total amount of blood in carrageenin-treated lungs (CARR) was unchanged compared with the control (CON; Table 1), but because of the increased lung weight, blood represented only 6% of the total wet weight rather than 12% as in the controls (Table 1). Glucocorticoid administration significantly re-

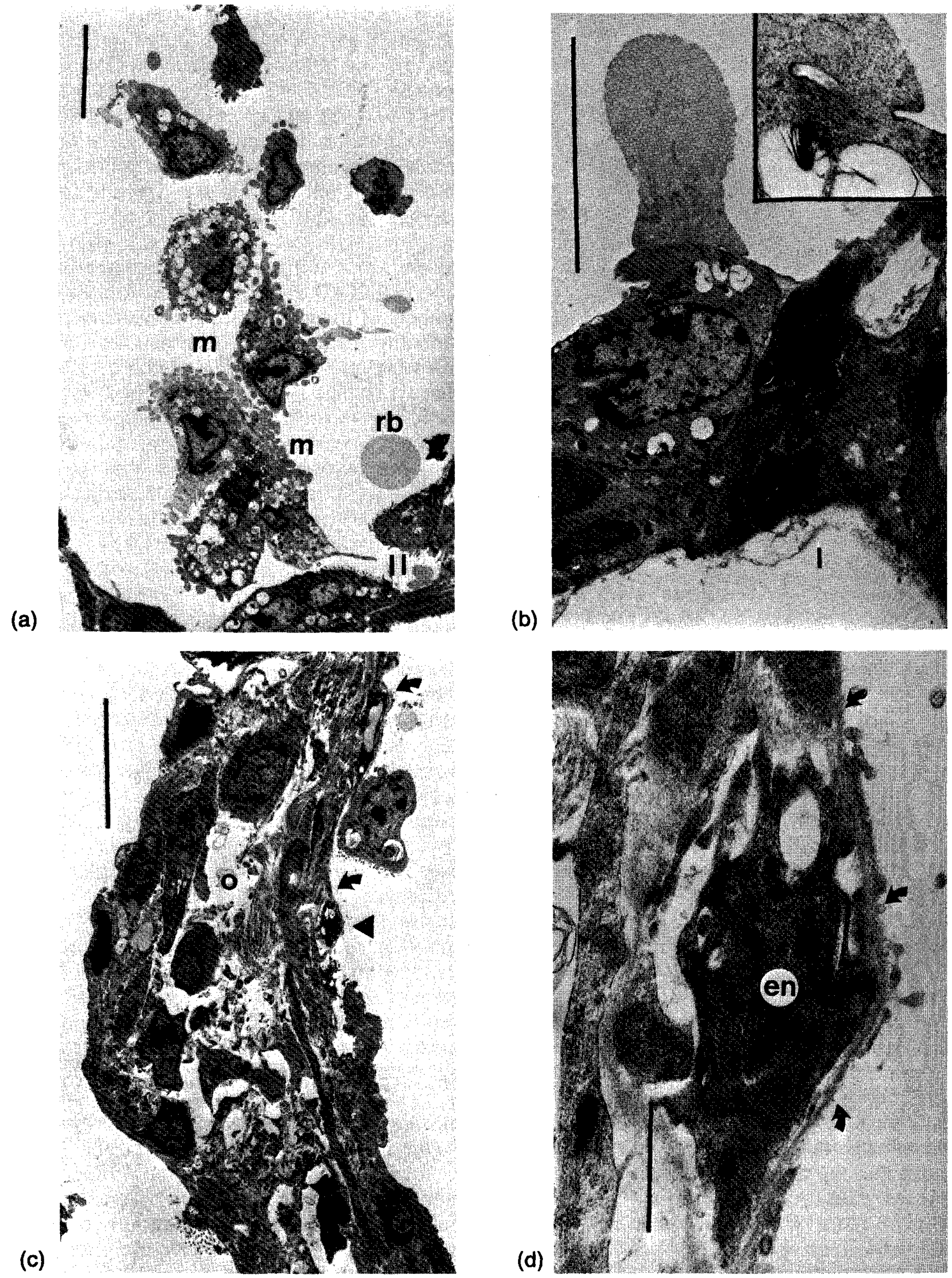

FIG. 3. Electron micrographs of lung sections from rats treated with carrageenin alone (CARR) and sacrificed 48 h after instillation. In each figure, the bar represents 10 microns unless otherwise stated. (a) Many foamy macrophages (m) and an electron lucent round body (rb) in the alveolar space; also in the section are two Type II epithelial cells (II) in close proximity (magnification ×2 000). (b) A Type II epithelial cell from which protrudes a bulbous extension; high magnification (×20 000) confirms cytoplasmic continuity with the Type II cell. The section also shows a damaged, vacuolated Type I (I) cell (magnification of main picture ×5 000). (c) Interstitial oedema (o) in close proximity to a region of denuded epithelial basement membrane (arrows), part of which (▲) is shown at higher magnification in Fig. 3(d) (magnification ×2 700). (d) Fragments of epithelium associated with a length of denuded basement membrane (arrow). Note also abnormal capillary endothelium (en) with increased density and cell shrinkage (magnification ×28 000; the bar represents 1 micron).

Table 1. Wet and dry lung and lung blood weights

| Group/ treatment | n | Wet lung wt (mg) (excluding wt of lung blood) | Dry lung wt (mg) (excluding wt of lung blood) | Lung blood wet wt (mg) | Lung blood (% total lung wet wt) |
|---|---|---|---|---|---|
| CON | 6 | $995 \pm 51^{b}$ | $116 \pm 9^{b}$ | $124 \pm 8$ | $11.3 \pm 2.4$ |
| BASE DEX | 7 | $806 \pm 34^{a}$ | $107 \pm 5$ | $102 \pm 9$ | $11.4 \pm 3.2$ |
| DEX | 6 | $705 \pm 24^{a}$ | $89 \pm 12^{a}$ | $93 \pm 4^{a}$ | $11.7 \pm 1.5$ |
| CARR | 6 | $1881 \pm 198^{c}$ | $231 \pm 24^{c}$ | $110 \pm 19$ | $5.9 \pm 3.1^{a}$ |
| CARR+DEX | 7 | $1144 \pm 83^{b}$ | $123 \pm 9^{b}$ | $107 \pm 15$ | $8.5 \pm 2.1^{d}$ |

CON, animals receiving no treatment;
BASE DEX, animals given dexamethasone (10 mg/l) in drinking water for 48 h prior to sacrifice;
DEX, animals given dexamethasone (10 mg/l) in drinking water for 96 h prior to sacrifice;
CARR, animals given 2.5 mg carrageenin intratracheally and sacrificed 48 h later;
CARR+DEX, animals given dexamethasone for 48 h prior to carrageenin exposure (2.5 mg intratracheally) and for a further 48 h after carrageenin exposure, after which they were sacrificed.
Data represent mean $\pm$ standard error of the mean; $^{a}$ < CON; $^{b}$ > DEX; $^{c}$ > all other groups; $^{d}$ < DEX; $p < 0.05$ in a Wilcoxon Rank Sum Test.

duced the lung water within 48 h (BASE DEX; Table 1) and the dry lung weight by 96 h (DEX). There was less blood in glucocorticoid-treated lungs (DEX) compared with the controls (CON; Table 1), but as a percentage of the total wet lung weight, it was unchanged (11–12%). The lungs of animals given both glucocorticoid and carrageenin (CARR + DEX) were intermediate between those given either stimulus alone, in terms of dry lung weight and lung water, and did not differ from controls (CON; Table 1). Likewise, the amount of blood in the lungs of rats given both carrageenin and dexamethasone (CARR + DEX) was not significantly different to any other group, but as a percentage of total lung weight, it was intermediate between carrageenin-treated animals (CARR) and those in the other treatment groups.

*Extracellular lipocortin 1 in bronchoalveolar lavage fluid:* Lc1 was detected by ELISA in BALF from all the animals investigated. Approximately 90% of the Lc1 in BALF from control (CON) rats was intact, as determined by Western blotting. Treatment with dexamethasone for 48 h (BASE DEX) or 96 h (DEX) was associated with a doubling of total Lc1 in BALF, which again, was over 90% intact. Carrageenin-treated rats (CARR) also had twice as much Lc1 in their BALF as controls, but over half of it was in the proteolysed form (Fig. 4). When both glucocorticoid and irritant were administered to the same animals (CARR + DEX), the total Lc1 concentration was approximately 10 times the control levels and was significantly greater than all the other treatments. However, as only 15% of this Lc1 was in its intact form, the concentration of intact Lc1 did not differ significantly from any other treatment group (Fig. 4). None of these changes could be explained by changes in BAL volume.

### Discussion

This study describes some of the morphological, cellular and biochemical changes occurring in the rodent lung following the intratracheal instillation of carrageenin. We observed an inflammatory response which was maximal at 48 h post-instillation and which was characterized by interstitial oedema and an influx of macrophages and PMN. Prior administration of an exogenous glucocorticoid greatly reduced the inflammation, confirming

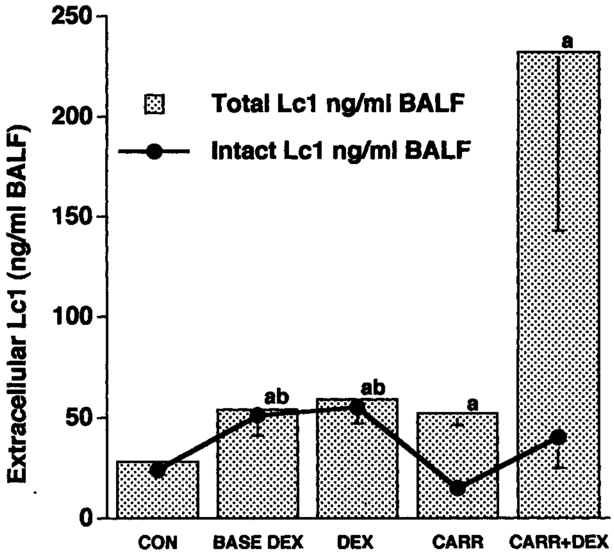

FIG. 4. Effect of treatment on total and intact extracellular lipocortin 1 concentration in bronchoalveolar lavage fluids. CON, animals receiving no treatment; BASE DEX, animals given dexamethasone (10 mg/l) in drinking water for 48 h prior to sacrifice; DEX, animals given dexamethasone (10 mg/l) in drinking water for 96 h prior to sacrifice; CARR, animals given 2.5 mg carrageenin intratracheally and sacrificed 48 h later; CARR+DEX, animals given dexamethasone for 48 h prior to carrageenin exposure (2.5 mg intratracheally) and for a further 48 h after carrageenin exposure, after which they were sacrificed.
$^{a}$total Lc1/ml > CON; $^{b}$intact Lc1/ml > CON; in each case, $p < 0.05$ in a Wilcoxon rank sum test for unpaired data. Data expressed as means $\pm$ standard error of mean; $n = 4$. Where no error bar is shown, the error is within the diameter of the symbol.

the hypothesis that carrageenin is essentially inert if the inflammatory response is subdued. Furthermore, dexamethasone was associated with an increase in extracellular levels of Lc1, an anti-inflammatory protein which may contribute to the actions of the glucocorticoid.

Historically, carrageenin has been used to provoke an inflammatory response in a wide range of tissues, including the lungs of rabbits, cats and rodents.[24–26] The effects of carrageenin on lung morphology in this study are similar to those reported previously,[25,27–30] namely, an early infiltration of PMN and macrophages, many of which phagocytosed carrageenin particles, plus epithelial cell damage and basement membrane denudation, followed by Type II cell hyperplasia. We have not found specific reference to the carrageenin-induced emergence of electron-lucent 'blebs' from Type II cells described in the current investigation. However, such a phenomenon has been described in a clinical context in a case of Type II cell adenocarcinoma and thus, is not necessarily a specific response to carrageenin,[31] but may be a nonspecific effect associated with epithelial cell damage and hyperplasia. In addition to confirming the morphological observations of previous authors, we have demonstrated that the response to carrageenin can be suppressed by pretreatment with dexamethasone.

In this study we found no change in the profile of circulating white cells after intratracheal carrageenin. This is in contrast to intraperitoneal administration in which there is a marked increase in the number of circulating PMN probably due to increased release from the bone marrow, perhaps combined with the lack of alternative routes of cell clearance from the peritoneal cavity[32] and a decline in monocytes.

Although an increase in oedema has been observed within 2 – 3 h after carrageenin in the rat paw,[10] in the lung we saw no morphological evidence of oedema at 4 h. However, by 48 h interstitial oedema was visible by EM (Fig. 3) and lung water was markedly increased (Table 1). Dexamethasone administration prevented the interstitial oedema and the accompanying epithelial damage and was also associated with a decline in macrophage numbers in the lung. This is in keeping with the findings of Heluy-Neto *et al.*[33] who studied the effects of glucocorticoid depletion (by adrenalectomy) on carrageenin-induced inflammation of the pleura, and reported that both the number of monocytes and the inflammatory exudate in the pleural cavity increased more following an adrenalectomy than a sham operation. Thus, together, these studies suggest that the activity of activated monocytes may cause oedema and exacerbate the inflammation, and that a major effect of the glucocorticoids is to down-regulate the actions of these cells.

One of the most intriguing features of the current model is that whilst the epithelial damage and interstitial oedema caused by carrageenin was clearly prevented by dexamethasone administration, this effect was accompanied by significant increases in both the number and percentage of lung PMN. This was observed despite the reduced number of circulating leukocytes and suggests that either the cells were actively recruited into the lung (by changes in chemotaxins or adhesion molecules) or were not cleared, perhaps because the PMN did not become apoptotic or because the reduced number of macrophages prevented removal of apoptotic PMN. The data further suggest that once inflammation is established, the PMN is less deleterious than initially. There is some evidence to support this view, suggesting that PMN may play a subtle role in modulation of the inflammatory and immune responses, and may even promote mechanisms of repair (reviewed in Reference 1). In addition, there is evidence that PMN are activated by cytokines and growth factors released from monocytes and macrophages such as GM–CSF and TNF. Hence, glucocorticoids may reduce PMN activity in the lung directly (for example, as indicated in Reference 34) or indirectly by reducing both the number of macrophages (Fig. 2) and their ability to release mediators which stimulate PMN.[35]

We hypothesize that the protective effect of the glucocorticoids may be due in part to the increase in extracellular Lc1 observed in this study. Lc1 probably exerts its anti-inflammatory activity by binding to cell surface receptors.[11] The increase in extracellular Lc1 observed in response to dexamethasone may have been caused by *de novo* Lc1 synthesis,[7] translocation to the cell surface[36] or a combination of both, and may have resulted in enhanced Lc1 activity within the lung. Interestingly, carrageenin administration was also associated with an increase in Lc1 in the epithelial lining fluid. We cannot exclude the possibility that carrageenin induced Lc1 indirectly, by stimulating a rise in circulating corticosterone. However, inflammatory stimuli such as paraffin oil[8] and endotoxin[37] have been shown to cause increases in Lc1 in glucocorticoid-depleted (adrenalectomised) rats, so it is possible that the increase was a direct effect of the inflammogen on resident or inflammatory cells. In carrageenin-treated animals, the predominant form of Lc1 was a 34 kDa species which lacks the N-terminal believed to confer functional specificity[6] and which was probably clipped by the action of neutrophil elastase.[14]

Reduction of intact Lc1 by proteolysis may feedback to stimulate further Lc1 synthesis and/or release, and this may explain the apparent synergy between carrageenin and dexamethasone with regard to extracellular Lc1 levels; the two stimuli may have increased extracellular Lc1 by different mechanisms. Although functional Lc1 was markedly reduced in the epithelial lining fluid of rats given carrageenin only, prior and concurrent administration of dexamethasone maintained intact (functional) extracellular Lc1 at the control level. The very high level of total Lc1 in animals given glucocorticoid and inflammogen suggests that release of this protein in its functional form earlier in the response may have contributed to the protective effect of dexamethasone. The anti-inflammatory mechanisms of Lc1 are complex and incompletely understood, but may include a dose-dependent reduction in interactions between IgG and Fc$\gamma$ receptors on PMN and monocytes,[38] an effect which may reduce the oxidant-producing capacity and other pro-inflammatory functions of these cells.

In summary, in this study we have used a model of carrageenin-induced pulmonary inflammation to investigate the possible anti-inflammatory mechanisms of action of dexamethasone. We conclude that an increase in extracellular Lc1 may be one mechanism by which this synthetic glucocorticoid controls pulmonary inflammation.

### References

1. Witko-Sarsat V, Deschamps-Latscha B. Neutrophil-derived oxidants and proteinases as immunomodulatory mediators in inflammation. *Mediators Inflamm* 1994; **3:** 257–273.
2. Swingle WW, Remmington JW. Role of adrenal cortex in physiological processes. *Physiol Rev* 1944; **24:** 89–127.
3. Lazar G, Agarwal MK. The influence of a novel glucocorticoid antagonist on endotoxin lethality in mice strains. *Biochem Med Metab Biol* 1986; **36:** 70–74.
4. Barnes PJ, Adcock I. Anti-inflammatory actions of steroids: molecular mechanisms. *Trends Pharmacol Sci* 1993; **14:** 436–441.
5. Bauer J, Lengyel G, Bauer TM, Acs G, Gerok W. Regulation of interleukin-6 receptor expression in human monocytes and hepatocytes. *FEBS Lett* 1989; **249:** 27–30.
6. Moss SE. The annexins. In: Moss SE, ed. *The Annexins.* London and Chapel Hill: Portland Press Research Monograph II, Portland Press, 1992; 1–9.
7. Goulding NJ, Godolphin JL, Sharland PR, *et al.* Anti-inflammatory lipocortin 1 production by peripheral blood leucocytes in response to hydrocortisone. *Lancet* 1990; **335:** 1416–1418.
8. Peers SH, Smillie F, Elderfield AJ, Flower RJ. Glucocorticoid- and non-glucocorticoid induction of lipocortins (annexins) 1 and 2 in rat peritoneal leucocytes *in vivo. Brit J Pharmacol* 1993; **108:** 66–72.
9. Vishwaneth BS, Frey FJ, Bradbury M, Dallman MF, Frey BM. Adrenalectomy decreases lipocortin-1 messenger ribonucleic acid and tissue protein content in rats. *Endocrinol* 1992; **130:** 585–591.
10. Cirino G, Peers SH, Flower RJ, Browning JL, Pepinsky RB. Recombinant lipocortin 1 has acute local anti-inflammatory properties in the rat paw oedema test. *Proc Natl Acad Sci USA* 1989; **86:** 3428–3432.
11. Goulding NJ, Luying P, Guyre PM. Characteristics of lipocortin 1 binding to the surface of human peripheral blood leucocytes. *Biochem Soc Trans* 1990; **18:** 1237–1238.
12. Fava RA, McKanna J, Cohen S. Lipocortin 1 (p35) is abundant in a restricted number of differentiated cell types in adult organs. *J Cell Physiol* 1989; **141:** 284–293.
13. Ambrose MP, Hunninghake GW. Corticosteroids increase lipocortin 1 in BAL fluid from normal individuals and patients with lung disease. *J Appl Physiol* 1990; **68:** 1668–1671.
14. Smith SF, Tetley TD, Guz A, Flower RJ. Detection of lipocortin 1 in human lung lavage fluid: lipocortin degradation as a possible proteolytic mechanism in the control of inflammatory mediators and inflammation. *Environ Health Persp* 1990; **85:** 135–144.
15. Ambrose MP, Hunninghake GW. Corticosteroids increase lipocortin 1 in alveolar epithelial cells. *Am J Cell Mol Biol* 1990; **3:** 349–353.
16. De Caterina R, Sicari R, Giannessi D, *et al.* Macrophage-specific eicosanoid synthesis inhibition and lipocortin-1 induction by glucocorticoids. *J Appl Physiol* 1993; **75:** 2368–2375.
17. Smith SF, Datta AK, Smith T, Tetley TD, Guz A, Flower RJ. Lipocortin 1 levels in lung lavage fluids collected from healthy volunteers before and after taking exogenous glucocorticoids. *Br J Pharmacol* 1994; **112:** 24P (abstract).
18. Smith SF, Flower RJ, Datta AK, Smith T, Tetley TD, Guz A. Effect of prednisolone on cellular lipocortin levels in lung lavage from healthy volunteers. *Am Rev Respir Dis* 1994; **149**(suppl): A334 (abstract).
19. Tetley TD, Rose FA, Richards RJ. Biochemical and cellular reaction of PVC paste polymers and latex following intratracheal instillation into rats. *Inflammation* 1981; **5:** 137–152.
20. Smith SF, Guz A, Cooke NT, Burton GH, Tetley TD. Extracellular elastolytic activity in human lung lavage: a comparative study between smokers and non-smokers. *Clin Sci* 1985; **69:** 17–27.
21. Van Kampen EJ, Zijlstra WG. Standardisation of hemoglobinometry. *Clin Chim Acta* 1960; **5:** 719–726.
22. Larsen GL, Webster RO, Worthen GS, Gumbay RS, Henson PM. Additive effect of intravascular complement activation and brief episodes of hypoxia in producing increased permeability in the rabbit lung. *J Clin Invest* 1985; **75:** 902–910.
23. Smith T, Smith SF, Buckingham JC. A competitive ELISA for the estimation of rat lipocortin 1. *Biochem Soc Trans* 1990; **18:** 1230–1231.
24. Trenchard D, Gardner D, Guz A. Role of pulmonary vagal afferent nerve fibers in the development of rapid shallow breathing in lung inflammation. *Clin Sci* 1972; **42:** 251–258.
25. Mitsuhashi T, Shimazaki M, Sugino S, Takeda T, Inariba H. Morphological studies on proliferation and desquamation of the alveolar lining epithelium in carrageenan-induced experimental pneumonia. *Tohuku J Exp Med* 1983; **140:** 319–330.
26. Gotoh M, Tsura S, Shinomiya N, *et al.* Functional changes of alveolar macrophages in carragheenan-induced aspiration pneumonia model mice. *Nat Immun* 1992; **11:** 345–355.
27. Wachtlova M, Chvalova M, Holusa R, Palecek F. Carrageenin-induced experimental pneumonia in rats. *Physiologia Bohemoslovaca* 1975; **24:** 263–266.
28. Mitsuhashi T, Kuwahara H, Ikura Y. Carrageenin-induced pulmonary emphysema of rabbit. *Tohuku J Exp Med* 1989; **157:** 163–176.
29. Bowers RR, Houston F, Clinton R, Lewis M, Ballard R. A histological study of the carrageenan-induced granuloma in the rat lung. *J Pathol* 1980; **132:** 243–253.
30. Bowers RR, Stapleton ME, Lew PD. An ultrastructural study of the macrophages of the carrageenan-induced granuloma in the rat lung. *J Pathol* 1983; **140:** 29–40.
31. Vasquez M, Sidhu GS. Surfactant production by neoplastic type II pneumocytes. *Ultrastruct Pathol* 1988; **12:** 605–612.
32. Tatsukawa K, Mitsuyama M, Takeya K, Nomoto K. Differing contributions of polymorphonuclear cells and macrophages to protection of mice against *Listeria monocytogenes* and *Pseudomonas aeruginosa. J Gen Microbiol* 1979; **115:** 161–166.
33. Heluy-Neto, Cunha FQ, Ferreira SH. *In vivo* mononuclear phagocyte migration: paradoxical effect of adrenalectomy. *Mediators of Inflamm* 1994; **3:** 275–279.
34. Fukushima K, Ando M, Ito K, Suga M, Araki S. Stimulus- and cumulative dose-dependent inhibition of $O_2^-$ production by polymorphonuclear leukocytes of patients receiving corticosteroids. *J Clin Lab Immunol* 1990; **33:** 117–123.
35. Schleimer RP. Effect of glucocorticosteroids on inflammatory cells relevant to their therapeutic applications in asthma. *Am Rev Respir Dis* 1990; **141:** S59–S69.
36. Solito E, Nuti S, Parente L. Dexamethasone-induced translocation of lipocortin (annexin) 1 to the cell membrane of U937 cells. *Br J Pharmacol* 1994; **112:** 347–348.
37. Smith T, Smith SF, Flower RJ, Buckingham JC. Endotoxin and the expression of lipocortins in central and peripheral tissues of the rat. *Br J Pharmacol* 1991; **104:** 218P (abstract).
38. Goulding NJ, Guyre PM. Lipocortin 1 binding to human leukocytes correlates with its ability to inhibit IgG interactions with Fc$\gamma$ receptors. *Biochem Biophys Res Commun* 1993; **192:** 351–358.

ACKNOWLEDGEMENTS. S.F.S. would like to thank the Trustees' Research Committee of Charing Cross & Westminster Medical School for financial support for this project.

**Received 28 February 1995; accepted in revised form 11 April 1995**
